# Complementary Feeding Practices and Childhood Malnutrition in South Africa: The Potential of *Moringa Oleifera* Leaf Powder as a Fortificant: A Narrative Review

**DOI:** 10.3390/nu15082011

**Published:** 2023-04-21

**Authors:** Hlengiwe Sokhela, Laurencia Govender, Muthulisi Siwela

**Affiliations:** Dietetics and Human Nutrition, School of Agricultural, Earth and Environmental Sciences, University of KwaZulu-Natal, Private Bag X01, Scottsville, Pietermaritzburg 3201, South Africa

**Keywords:** dietary diversity, food-based interventions, indigenous crops, malnutrition, *Moringa oleifera*, weaning foods

## Abstract

Poor complementary feeding is a common practice in developing regions, including South Africa (SA), and is one of the main contributing factors to childhood malnutrition. This paper reviews the literature on complementary feeding practices in SA and the potential of fortifying home-prepared complementary foods with *Moringa oleifera* to improve their nutritional composition. Studies that investigated complementary feeding practices, indigenous crops, nutritional benefits of *Moringa oleifera*, and the use of MOLP as a fortificant both locally and globally were included in this review. In SA, maize meal and commercial cereal are the most commonly used complementary infant foods. The diet consumed by children from vulnerable households commonly has insufficient nutrients. Foods consumed are generally high in starch and low in other essential nutrients, including good-quality protein. Impoverished individuals consume poor-quality foods as they are unable to afford a diversified diet with food from different food groups, such as protein, fruits, and vegetables. In SA, various programs have been implemented to reduce the incidence of childhood malnutrition. However, childhood malnutrition remains on the rise. This shows a need for complementary food-based strategies that can be implemented and sustained at a household level. This can be conducted through the use of accessible indigenous crops such as *Moringa oleifera*. *Moringa oleifera* contains essential nutrients such as proteins, amino acids, vitamins, and minerals. Therefore, it could possibly be used as a home-prepared complementary food fortificant to enhance nutritional composition. Before complementary foods can be fortified with *Moringa oleifera*, popular home-prepared complementary foods must be identified.

## 1. Introduction

Like other developing regions, South Africa (SA) faces the burden of malnutrition [1] in the form of undernutrition, a public health concern affecting vulnerable population groups, especially children under the age of five years [2]. The trend in SA follows that in developing countries [2]. In 2020, 22% and 6.7% of children under five years worldwide were stunted and wasted, respectively [2]. Similarly, in SA in 2016, 27.4% of children under five years were stunted, 6% were underweight, 2.5% were wasted, and 13% had micronutrient deficiencies [3]. Additionally, in KwaZulu-Natal (KZN), a province in SA, it was found that in 2017, stunting affected 28.5% of children under five years [4]. These statistics show that stunting is the most common form of malnutrition in South African children under five years [5]. 

Several factors are associated with the escalating rates of malnutrition, especially undernutrition in SA. Still, some major contributing factors are food insecurity, poor feeding practices for infants and young children, childhood illnesses, and poor access to water and sanitation [6]. As mentioned earlier, children under five years are the most vulnerable to undernutrition; children must receive adequate nutrition in the first 1000 days of life, as this is a critical period for growth and development [7]. Not all individuals can afford adequate food during pregnancy and lactation [7]. Furthermore, complementary feeding can be suboptimal due to poor access to nutritious foods [8]. Poor complementary feeding practices primarily cause childhood malnutrition [8]. The World Health Organization (WHO) recommends initiating complementary feeding at six months to prevent malnutrition and provide adequate energy, protein, and micronutrients. In SA, early introduction of solids before six months and insufficient consumption of protein-rich foods by children between the ages of 6–24 months are common practices that lead to protein-energy malnutrition (PEM) and micronutrient deficiencies [9]. Therefore, programs and/or strategies must be implemented to address childhood malnutrition.

The government in SA has implemented various programs and/or strategies, such as breastfeeding promotion, growth monitoring and promotion, vitamin A supplementation, and food fortification, to help reduce malnutrition and improve the nutritional status of children [8]. However, these programs have had various challenges that have hindered their implementation or access [10]. COVID-19 increased the limitation of access to these programs and negatively affected access to basic nutrition and healthcare services [10]. As a result, malnutrition cases are expected to rise [11]. More feasible home-based strategies are needed to reduce malnutrition, especially during challenging economic times, as was the case during the COVID-19 pandemic [11]. Affordable, accessible, and sustainable strategies are needed to complement existing strategies to reduce malnutrition and improve the nutritional status of vulnerable populations, especially children [11]. Therefore, other sustainable and accessible strategies, such as improving the nutritional content of home-prepared complementary foods to combat malnutrition at a household level, are required. Nutrient-rich plant material obtained from accessible, affordable, and locally adapted plant species could be used as a fortificant in home-prepared complementary foods.

*Moringa oleifera* is native to India and thrives well in tropical and subtropical regions in different parts of the world [12,13]. South Africa has suitable land conditions that allow for the growth of this crop. Thus, it is produced in most provinces in SA [14]. It is widely cultivable, as it can grow in less fertile soil, and its growth is rarely affected by drought [12,13]. Several studies have shown that *Moringa* leaves are rich in health-promoting bioactive substances and several nutrients, including protein and micronutrients [12]. Therefore, there is a growing interest in promoting the utilization of *Moringa* leaves as a health and nutritional ingredient to address malnutrition and related health conditions. *Moringa* leaves are processed into *Moringa* Leaf Powder (MOLP), now widely available in formal and informal markets in SA [14]. However, most South Africans are unaware of the health and nutritional benefits of *Moringa* leaves [13]. Global and local studies have been conducted to determine consumer acceptance of *Moringa* in complementary foods. While incorporating MOLP in nutrient-deficient foods improves the nutritional composition [15,16], the MOLP might decrease consumer acceptability as it would likely negatively affect the taste, colour, and aroma, as found in previous studies [15]. Therefore, for MOLP to be used as a fortificant by the target community, popular home-prepared complementary foods whose consumer acceptance would not be negatively affected by the incorporation of MOLP should be identified. Therefore, this narrative review aims to provide a comprehensive picture of the complementary feeding practices in SA, specifically within KZN province, and evaluate the potential of including MOLP as a fortificant to improve the nutritional composition of home-prepared complementary foods.

## 2. Methodology

This paper reviewed the literature on complementary feeding practices and home-prepared complementary foods that could be fortified with MOLP to improve their nutritional composition. Studies that investigated complementary feeding practices, indigenous crops, the nutritional benefits of *Moringa oleifera*, and the use of MOLP as a fortificant, both locally and globally, were included in this narrative review. The inclusion criteria used were studies written in English, peer-reviewed studies, government reports, and policies published from 2014 onwards. Only indispensable older literature (e.g., the literature on basic principles, concepts, and facts) and articles between 2007–2022 on complementary feeding practices in SA were also used. Relevant literature sources were obtained using several academic search engines, including PubMed, Science Direct, and Google Scholar. A comprehensive literature search was conducted using the following search words: “complementary feeding”, “dietary diversity”, “food-based interventions”, “homemade complementary foods”, “indigenous crops”, “introduction to solids”, “malnutrition”, “*Moringa*”, “weaning foods”. Abstracts were read, and manuscripts were selected as per the inclusion criteria.

## 3. Malnutrition in Children: Undernutrition in South Africa

Undernutrition, a form of malnutrition, refers to deficiencies or imbalances in an individual’s daily requirements for energy and essential nutrients from the diet [17]. As alluded to earlier, childhood undernutrition can present as stunting, underweight, wasting, and/or micronutrient deficiencies. Undernutrition can be further classified as severe acute malnutrition (SAM), moderate acute malnutrition (MAM), and not acutely malnourished but at risk (NAM but at risk) [17].

Stunting is a chronic form of undernutrition; it increased by 2.5% between 2008–2016 in SA [5,7,8]. Furthermore, the second highest percentage (28.5%) of stunting was found in the KZN province in SA, and children residing in rural areas were the most affected by stunting (29%) in comparison to their urban counterparts (26%) [3]. Stunting is irreversible after the age of two [18,19]. It has several negative effects, such as poor growth and development and irreversible physical and cognitive damage caused by persistent nutritional deprivation [20,21,22]. This emphasizes the need for adequate nutrition in the first 1000 days of life.

The statistics from the latest published South African national study conducted in 2016 indicated that 6% of South African children were underweight. At the same time, wasting affects 2.5% of South African children [7]. Furthermore, 2.5% of children under five from the KZN province are affected by wasting [3]. Wasting is caused by hunger, insufficient intake of adequate quantity and quality of food, and regular incidence of childhood illnesses that affect the nutritional status, or a combination of these factors [17,23]. Wasting can result in childhood mortality if not prevented, identified, or treated accordingly [3], reemphasizing the importance of good nutrition, especially during complementary feeding.

Protein-energy malnutrition (PEM) is a form of malnutrition affecting children under five in Africa and other developing countries [8]. PEM can be defined as the consumption of a diet by children that contains insufficient protein and energy, which are needed for normal growth and development [24]. In addition to PEM, children may also be affected by micronutrient deficiencies (e.g., iron, zinc, iodine, and vitamin A), which can also cause an irreversible effect on growth and development [25]. PEM and micronutrient deficiencies manifest early in children between 6–24 months [25]. Poor nutrition and infectious diseases can cause PEM and micronutrient deficiencies in children [20], showing the importance of optimal nutrition in early childhood.

There are several causes of undernutrition in SA, namely poverty, food insecurity, lack of access to a variety of nutritious foods, inadequate infrastructure, lack of economic resources and access to healthcare facilities, lack of education and knowledge of appropriate feeding practices, and infectious diseases [25,26]. Globally and in SA, the burden of undernutrition continues to increase despite social and economic development [27].

Many vulnerable individuals rely on social grants to purchase food and necessities. Social grants are not sufficient to provide individuals with nutritiously balanced meals. For example, a child support grant in SA is R500/month ($27.54) [28], and a basic 28-item food basket in SA costs R1148.38 ($63.25) [29]; this shows that the affordability of foods that contain essential nutrients is far from reach for many vulnerable individuals. Insufficient dietary intake of essential nutrients from foods can cause diseases, leading to childhood undernutrition and even death [30]. In SA, consuming a monotonous diet negatively affects the nutritional status of children under five years [8]. A monotonous diet lacks essential nutrients, such as protein, vitamins, and minerals, and is made up mostly of starchy foods [8,31]. Therefore, insufficient intake of essential nutrients due to consuming a diet that lacks variety or food from different food groups may contribute to childhood undernutrition [8], thus stressing the importance of strategies to assist in reducing and preventing undernutrition.

## 4. Strategies in Place to Combat Childhood Malnutrition and Challenges in the South African Context

The interventions currently being implemented in SA to address childhood undernutrition include breastfeeding promotion [32], growth monitoring and promotion (GMP) programs [8], vitamin A supplementation programs [33], and food fortification [34].

Infants need to be exclusively breastfed for the first six months and thereafter introduced to solids, as breastmilk alone is not sufficient to meet the infant’s nutritional needs. Breastfeeding is continued with complementary foods for up to two years and beyond [35]. When comparing breastfeeding rates, it is noteworthy that rural areas have higher rates than urban areas [36]. However, a common universal practice of mixed feeding has been noted in the first six months due to several factors. In SA, suboptimal breastfeeding rates are influenced by easy access to infant formula [37]. Working women do not receive adequate support to continue breastfeeding [38]. Only 12% of countries worldwide provide adequately paid maternity leave, thus resulting in lactating women wanting to return to work to earn income [39]. This, in turn, can lead to the cessation of breastfeeding and the early commencement of complementary foods [39]. This is a risk factor for undernutrition, as early initiation of complementary foods significantly impacts the infant’s growth, development, and survival [40], which are assessed during the GMP clinic visits.

GMP consists of regular growth assessment, where appropriate referrals are made if necessary, e.g., referral to nutrition services if the infant is underweight. The GMP program can potentially reduce childhood undernutrition through early diagnosis and referral to relevant services [41]. GMP programs have various challenges, particularly in developing countries like SA, including limited availability of equipment used for anthropometric assessments, poor recording on growth charts, poor attendance of patients at healthcare facilities for GMP, and remote patients being unable to afford transportation to healthcare centres [42]. Regardless of regular GMP attendance, some caregivers still lack knowledge of appropriate infant feeding practices and/or lack support at the household level to implement the recommended infant and young child feeding practices [42]. Additionally, in SA, healthcare workers face challenges that prevent the implementation of the GMP, such as work overload, vaccine shortages, insufficient staff training on GMP, and inadequate supply of health booklets used to record immunizations and growth trends [43]. As in other developing countries, in SA, the vitamin A supplementation program is part of the GMP program; infants are routinely supplemented with vitamin A during their GMP clinic visits to prevent deficiencies that can negatively affect growth.

Vitamin A deficiency (VAD) is a major public health problem in low- and middle-income countries [44], including SA. VAD can cause blindness and increase the risks of childhood death and illness from infections such as measles and diarrheal diseases [45]. Addressing VAD can improve disease resistance and reduce mortality in children under five, as vitamin A supplementation supports growth and combats infections [46]. Although SA has a vitamin A supplementation program, VAD is still a concern, as 44% of children under five years have VAD, which is higher than the statistics from the 1994 South African Health and Examination Survey, which found 33.3% had VAD [47].

This emphasizes the need for accessible and sustainable strategies such as food fortification with essential nutrients and existing strategies to reduce undernutrition.

Food fortification is the addition of essential micronutrients, such as trace elements, vitamins, and minerals, to processed foods to improve their nutritional quality [34]. Food fortification is used to reduce micronutrient deficiencies such as vitamin A, vitamin B, vitamin D, and iron [48]. It is a cost-effective food-based approach that can be used to reduce micronutrient deficiencies [48,49]. Most commonly consumed foods in developing countries are used as vehicles for fortification [50]. These include oil and fats, milk, sugar, salt, rice, wheat, and maize [50].

In SA, maize meal and wheat flour are fortified with vitamin A, thiamine, riboflavin, niacin, pyridoxine, folic acid, iron, and zinc [51]. The public health benefit of food fortification includes prevention and/or correction of micronutrient deficiency and improving nutritional status in a specific population group [52]. Although fortified foods are available in SA, they are not affordable or accessible to most vulnerable groups, thus increasing their risk for micronutrient malnutrition [51]. This shows a need for affordable food-based strategies to support existing strategies to reduce undernutrition in early childhood, especially in the complementary feeding phase.

## 5. Complementary Feeding Practices in South Africa

It is important to promote good growth and prevent undernutrition during the first 1000 days of life, which is the period from when the child is conceived to two years of age [53]. As mentioned earlier, infants must be exclusively breastfed for the first six months of life, followed by the introduction of safe, nutritious complementary foods and breastmilk [54]. According to the WHO, complementary feeding must be timely (introduced not too early or too late), adequate (provide sufficient energy and nutrients as per individual’s requirement), safe (hygienic practices must be observed during meal preparations, storage, and feeding), and age-appropriate [54]. Complementary food should contain sufficient amounts of energy, macronutrients (protein, fats, and carbohydrates), and micronutrients (e.g., zinc, iron, calcium, folate, and vitamins A, B, and C) needed to ensure that the recommended growth and development are achieved [55]. Poor complementary feeding practices include giving foods with inadequate nutrients, starting solids too early or too late, and/or infrequent feeding. In SA, children between 6 and 24 months consume a minimal amount of food with protein, which leads to PEM and deficiencies of essential nutrients [9]. Early introduction of complementary foods can result in poor breastfeeding and infant feeding practices [55]. Increased choking risks, food allergies, and reduced breast milk intake are the dangers associated with the early introduction of solids [55]. In SA, the introduction of solids before six months is common [9]. Late introduction of complementary foods can result in developmental delay and increased risk of malnutrition, leading to stunting, wasting and/or being underweight [55,56].

There are two types of complementary foods: commercial and home-prepared complementary foods [55]. Commercial complementary food is bought from the supermarket and includes food items such as iron-fortified infant cereal and commercially prepared fruits, vegetables, and meat [55,57]. Home-prepared complementary food is food prepared at a household level by a caregiver using traditional methods [55]. Commercially fortified foods are often not accessible to vulnerable populations due to affordability. Hence, home-prepared complementary foods are commonly used by vulnerable populations [55]. In developing countries, complementary foods are often home-prepared from staple crops, as commercial complementary food are expensive [58]. However, the challenge with home-prepared complementary foods is poor dietary quality [59]. Home-prepared complementary foods in sub-Saharan African countries usually contain starchy staples of poor nutritional quality [58]. In SA, home-prepared maize meal porridge and commercial infant cereal are the most common complementary foods [9]. In SA, maize meal is the most common first food introduced to infants, but the popularity of commercial infant cereal has also increased over the years [9].

In SA, some studies that have investigated complementary feeding practices. In 2022, Sayed and Schönfeld (2022) conducted a cross-sectional study using 184 mothers from KZN. The study aimed to ascertain whether the nutrient requirements of 6–11-month-old infants could be met with a food-based approach and to identify which nutrients from food were consumed in insufficient amounts. The results revealed that iron, zinc, and calcium were the nutrients that were not provided in sufficient amounts in the diet of infants from the study group [60]. Another study conducted by Chakona (2020) of 84 Eastern Cape mothers in their maternal and infant/young child dietary diversity and breastfeeding practices found that 56% of mothers perceived meal porridge as the type of food that should be given to a child for growth. In this study, 35%, 32%, and 12% of mothers perceived vegetables, fruits, and meat as essential for growth. The most commonly used complementary food item given to infants by caregivers (57%) was maize meal porridge [61]. A longitudinal study of pregnant women during their second trimester from rural and urban clinics in Western Cape, SA, found that 19% of infants were given complementary foods at four months.

Moreover, the study found there was high consumption of processed meats (56%) and inappropriate foods such as fruit juice (82%), soft drinks (54%), and refined sugar (51%) at one year [62]. A descriptive study using a structured questionnaire was conducted by Seonandan and McKerrow (2016) of mothers of infants up to the age of five years and healthcare professionals in 12 state hospitals in KZN province, SA. These authors found 84% of infants below six months of age were introduced to complementary feeding [63]. Similarly, a prospective cohort study of 168 mothers living in KZN found 73% of infants were given complementary foods at 14 weeks [64]. Likewise, a descriptive and exploratory study conducted in Limpopo of 185 mothers with infants 12 months old and younger in a primary healthcare clinic found 15% of mothers started complementary feeding before two months and 43.2% at three months. The weaning food most of the mothers gave was soft maize meal porridge, which had been introduced before four months of age [65]. Additionally, a cross-sectional survey and an unquantified food frequency questionnaire conducted by Cape, Faber and Benade (2007) in KZN found the first solids were maize meal porridge (55%), infant cereal (32%), and ready-to-eat bottled baby foods (9%).

The above studies show that most infants are fed poor-quality complementary foods, with maize meal porridge being the most common complementary food item given to infants. Maize is a staple that most vulnerable households can afford and is consumed by infants as the main complementary food in SA. In SA, maize is fortified with vitamin A, thiamine, riboflavin, niacin, pyridoxine, folic acid, iron, and zinc to prevent deficiencies. It must be consumed in specific amounts to meet the nutritional requirements; it also does not contain all essential nutrients, such as protein, which promotes growth and development. To increase the dietary diversity of maize, it must be eaten with other nutrient-dense food items, such as meat and/or vegetables, which vulnerable populations might not be able to afford. Therefore, home-based strategies that can be used to improve the nutritional composition of homemade complementary foods should be considered, such as using MOLP as a fortificant.

## 6. The Potential Use of MOLP as a Fortificant

### 6.1. Background

*Moringa* is a tree native to India. It belongs to the *Moringaceae* family and is the most cultivated species of the genus *Moringa* [66]. *Moringa* is widely cultivable in different parts of the world and can thrive in different environmental conditions, such as severe drought and mild frost conditions [14,67]. *Moringa oleifera* was first introduced in SA to rural communities in the Limpopo province as a cultivable crop. Since then, production and utilization have increased in different parts of SA [14]. *Moringa* has a high nutritional value of both macronutrients and micronutrients [12]. Therefore, *Moringa oleifera* could be used as a source of nutrition to combat undernutrition in children [66].

### 6.2. Nutritional Composition of Moringa

*Moringa oleifera* contains protein, amino acids, vitamins (A, B, and C), and minerals (calcium, phosphorus, and iron), which can be used to combat undernutrition and improve food security through its incorporation into food [68,69]. All parts of *Moringa oleifera* store essential nutrients [12]. The nutritional composition of *Moringa* leaves and leaf powder is presented in Table 1 [70,71]. *Moringa oleifera* leaves are usually used as a food ingredient in a powder called *Moringa oleifera* leaf powder (MOLP) [72]. *Moringa* leaves are converted into MOLP by drying fresh *Moringa* leaves [72]. However, drying the *Moringa* leaf into powder affects its nutritional composition [72]. It has been shown that dry *Moringa* leaves and MOLP have a higher concentration of energy, protein, and fat content when compared to fresh *Moringa* leaves; however, the concentration effect does not prevent the possible loss of a certain proportion of specific nutrients during processing [72].

*Moringa* contains essential vitamins (A, B, and C) and minerals (calcium, phosphorus, and iron). This includes the SA *Moringa* ecotype [14]. *Moringa* also has high vitamin E and beta-carotene content. It provides seven times more vitamins than oranges, ten times more vitamin A than carrots, seventeen times more calcium than milk, nine times more protein than yoghurt, and twenty-five times more iron than spinach [12]. Minerals such as calcium, iron, potassium, copper, and zinc found in *Moringa* are essential in preventing and treating malnutrition and micronutrient deficiencies. These minerals are essential for physiological growth and development [12].

*Moringa* is a good source of protein and other essential nutrients. Plant-based protein alternatives such as *Moringa oleifera* can be used as a treatment or preventative measure for undernutrition [13,73]. Essential amino acids are obtained through the consumption of food items such as eggs, poultry, fish, and red meat. This is a problem for the vulnerable population who cannot afford to purchase meat-based food sources of protein. The amino acid levels of threonine, methionine, isoleucine, lysine, and valine present in *Moringa* leaves are almost identical to those found in meat products [74]. Plant-based protein from crops such as *Moringa* can be a more affordable and accessible source of protein when compared to animal-based protein [74]. The protein content (dry matter) of *Moringa* in the form of dry leaves (29.4%) and leaf powder (27.1%) was found to be higher when compared with other plant-based leaves such as Mulberry leaves (20.9%), Anchote leaves (21.6%) and Alfalfa leaves (18.1%) [75].

### 6.3. Utilization of Moringa Oleifera Leaves as a Food Source—Consumer Acceptability

In SA, the government has implemented various activities to support *Moringa* production [14,76]. These activities include the inclusion of *Moringa* on the list of medicinal plants in SA, offering financial support for *Moringa*-based development activities, and allowing research to be conducted on the inclusion of *Moringa* in the daily diet of school-going children [14]. The different parts of the *Moringa* plant can be consumed in a number of ways. In SA *Moringa* is consumed as seeds (used as ground nuts and as spices) and leaves (leaf powder is added in salads or cooked as a vegetable or as a *Moringa* tea) [14].

Currently, *Moringa* leaves are being processed for use as a commercial food fortificant in the form of MOLP. Various global and local studies have been conducted to determine consumer acceptability and nutritional composition of food items using *Moringa* as a fortificant. This has both advantages and disadvantages. The advantages and disadvantages of using *Moringa* as a fortificant found in different studies between 2014–2022 are presented in Table 2.

According to the research findings presented in Table 2, fortification with *Moringa* can improve the protein and other essential nutrient content of foods when it is used as a fortificant. At higher substitution levels, the nutrient profile of the fortified product improves. Based on the fact that the nutritional content improves when *Moringa* is added, consumption could help in reducing undernutrition [77]. However, even if a product has a good nutritional value, it will not positively impact one’s health if it is not well accepted. Most studies indicated that a *Moringa*-fortified product’s acceptability decreases as the substitution level increases. *Moringa oliefera* leaves contain chlorophyll, giving them a dark green colour [78]. When MOLP is added to, e.g., white maize porridge, it turns the porridge from a cream-white colour to a greenish colour, which can be undesirable. Furthermore, *Moringa* can make a product bitter if too much is added, giving the product an unpleasant taste. A review by Boateng et al. indicated that the optimal substitution for improved nutritional content and sensory acceptability is 10%. Any substitution higher than 10% has poor consumer acceptability [79]. Another study indicated that 5% substitution is the optimal level to use for the fortification of food products [77]. Another way to improve consumer acceptability would be to add another food item to mask *Moringa*’s taste and/or colour.

### 6.4. Effectiveness of Moringa as a Supplement to Prevent Childhood Undernutrition

In developing countries such as India and Nigeria, *Moringa* has been included in children’s and breastfeeding women’s diets as an intervention to prevent childhood malnutrition [14,80]. Table 3 indicates studies conducted between 2014–2022 on the effectiveness of *Moringa* as a supplement in preventing and treating child malnutrition. All except for one study showed the positive effects of *Moringa* supplementation. There was weight gain, improved haemoglobin levels, reduced anaemia, and improved overall nutritional status. From the studies presented, the dose and duration of *Moringa* supplementation are important considerations for ensuring nutritional benefits.

**Table 2:** The advantages and limitations of using MOLP as a fortificant in foods that can be used for complementary feeding.

Author/s	Aim	Method	Study Area	Vehicle for Fortification	Advantages	**Limitations**
Olusanya et al. (2020) [16]	To investigate the effects of MOLP on the nutritional composition of and consumer acceptability of mahewu.	2%, 4%, and 6% *w*/*w* levels of MOLP were added to the mahewu. Fifty-two untrained panellists participated in the sensory evaluation.	KZN, SA	Mahewu	The total mineral (calcium and iron), fat, and fibber increased.	The colour and aroma acceptability decreased with the addition of MOLP to the mahewu.
Ntila et al. (2019) [81]	To determine the caregiver’s acceptability and perception of soft white maize porridge modified with MOLP.	Cross-sectional (sensory evaluation: 60 mothers and focus groups) 1%, 2%, and 3% *w*/*w* levels of MOLP were added to the soft white maize porridge.	Gauteng and Limpopo, SA	Soft white maize porridge	All caregivers were willing to use *Moringa* in complementary foods, provided they would be educated on how to process it.	Consumer acceptability decreased as the level of MOLP increased. Caregivers indicated that the MOLP porridge was bitter and would not be accepted by their children.
Boateng et al. (2018) [82]	To investigate the acceptability of complementary foods fortified with MOLP.	MOLP was given to infants as part of a *koko* or sprinkled on foods. A total of 36 infant–mother pairs were studied.	Ghana	*Koko* (a fermented maize porridge)	Authors found that *Moringa* in the cereal blend and sprinkled on food was well accepted by caregivers.	-
Netshiheni et al. (2018) [83]	To determine the effect *Moringa oliefera* and termite powder on the nutritional and sensory properties of instant maize porridge.	The inclusion of *Moringa oliefera* and termite powder in instant maize porridge using different treatments was considered using a completely randomised design. Sixty untrained panellists rated the appearance, texture, taste, aroma, and overall acceptability of fortified instant maize porridges	Venda	Instant maize porridge	Results indicated the fortified porridge had a higher protein content than the unfortified porridge. Zinc, iron, Calcium, and magnesium were also higher in the fortified samples.	The untrained panel rated the control sample as better than the fortified samples.
Abioye and Aka (2015) [84]	To identify the effects of *Moringa* leaf fortification on the nutritional value and consumer acceptability of maize ogi.	Cross-sectional study. The ogi produced from maize was fortified with *Moringa* leaf substitution levels of 10% and 15%.	Nigeria	Maize-ogi	Improvement in nutritional and sensory quality. Increased protein, mineral content, and crude fibre. Ten percent *Moringa* substitution in maize-ogi is the optimal substitution for consumer acceptance.	Swelling capacity decreased with an increase in the level of *Moringa* substitution. The sensory properties of the 15% substitution were not comparable to the unfortified sample.

## 7. Conclusions

This narrative review aimed to discuss malnutrition in the form of undernutrition in children, the strategies in place in SA to combat childhood malnutrition and its challenges, complementary feeding practices in SA, specifically within KZN, and the possibility of including MOLP as a fortificant to improve the nutritional composition of home-prepared complementary foods. Despite different programs implemented by the government in SA to combat malnutrition, children under five years are still affected by malnutrition. Poor complementary feeding practices in SA are common and are the main contributing factor to childhood malnutrition. In SA, popular complementary foods are maize and commercial cereals. However, vulnerable populations have access to maize fortified with only some nutrients and lacking in other essential nutrients such as protein. This is needed for infant growth, as deficiency can result in malnutrition, which can have an irreversible effect, as discussed earlier in this review.

There is a need to implement home-based food strategies to improve the nutritional composition of popular home-prepared complementary foods. A possible food-based approach could be to incorporate MOLP into popular home-prepared complementary foods. *Moringa* is a nutrient-dense plant that can potentially improve children’s nutritional status, especially in the complementary feeding phase. However, *Moringa* has been associated with several undesirable sensory properties, thus hindering its consumer acceptability. Therefore, there is a need for further investigation to develop a suitable fortified complementary food with optimal substitution and improved nutritional composition. Before this can be done, home-prepared complementary foods suitable for the incorporation of MOLP should be identified for the target population. Further research needs to be conducted to improve the physical appearance and taste of *Moringa*-fortified foods to improve consumer acceptability. Moreover, studies could look at adding more than one food item to mask the colour and enhance the taste.

## Figures and Tables

**Table 1:** The nutrient composition of *Moringa oleifera* fresh leaves, dry leaves, and leaf powder.

Nutrients	Fresh Leaves	Dry Leaves	Leaf Powder
Energy (cal)	92	329	205
Protein (g)	6.7	29.4	27.1
Fat (g)	1.7	5.2	2.3
Carbohydrates (g)	12.5	41.2	38.2
Fibre (g)	0.9	12.5	19.2
Vitamin B1 (mg)	0.06	2.02	2.64
Vitamin B2 (ng)	0.05	21.3	20.5
Vitamin B3 (mg)	0.8	7.6	8.2
Vitamin C (mg)	220	15.8	17.3
Vitamin E (mg)	448	10.8	113
Calcium (mg)	440	2185	2003
Magnesium (mg)	42	448	368
Phosphorus (mg)	70	252	204
Potassium (mg)	259	1236	1324
Copper (mg)	0.07	0.49	0.57
Iron (mg)	0.85	25.6	28.3
Sulphur (mg)	-	-	870

All values are in 100 g per plant matter.

**Table 3:** Studies on the effectiveness of *Moringa* as a supplement in the prevention and treatment of undernutrition in children.

Author/s	Materials and Methods	Aim	Area	Subjects	**Main Findings**
Brar et al. (2022) [85]	Systematic review	To assess the impact of *Moringa* leaf supplementation in humans and animals on the outcomes of iron level, vitamin A status, the measures of growth, and/or breastmilk production.	-	One-hundred forty-eight unique studies, 33 were included (7 human studies and 26 animal studies).	In humans, *Moringa* at higher (14–30 g/day), not lower (<10 g/day) doses improved haemoglobin (Hgb) in children with iron deficiency anaemia; *Moringa* (0.5 g/day) also increased breastmilk volumes.
Yadav et al. (2022) [86]	Randomised controlled trial	To assess the effect of *Moringa oliefera* leaf powder supplementation on children with Severe Acute Malnutrition (SAM) during facility-based care and home-based care.	Gwalior District of Central India	One hundred children in the age group of 7–59 months admitted between November 2019 and October 2020 who fulfilled the World Health Organization (WHO) recommended criteria for identification of severe acute malnutrition were included in the study.	The use of MOLP supplementation resulted in an improved weight gain and reduction in severe wasting at the end of two months.
Manzo et al. (2021) [87]	Randomised double-blind clinical trial	To analyse the impact of supplementation of *Moringa oliefera.*	Niger	Four hundred children with moderate acute malnutrition (MAM) aged 6 to 59 months were admitted to outpatient nutritional recovery centres.	There was no difference in average weight gain or mid-upper arm circumference and size between the children who were supplemented with *Moringa* and those who were not supplemented with *Moringa*.
Shija et al. (2019) [88]	Community-based interventional study	To investigate the effect of *Moringa oliefera* leaf powder supplementation in reducing anaemia among children younger than two years.	Kisarawe District, Tanzania	Ninety-five anaemic children were followed for six months.	Increasing the amount and time of using *Moringa oliefera* supplementation significantly reduced cases of anaemia.
Srikanth et al. (2014) [80]	The nutritional intervention was given in *Moringa Oleifera* leaf powder 15 g twice daily for two months. Reassessment of the nutritional status was done following the intervention.	To identify children with Protein Energy Malnutrition. To give nutritional intervention in the form of *Moringa oleifera* powder to the children for two months. To reassess the nutritional status after the nutritional intervention at the end of two months.	Rural Area in Bangalore, India	Sixty children with grade I and grade II protein energy malnutrition.	Seventy percent of children with grade II PEM improved to grade I, and 60% with grade I PEM showed significant (*p* < 0.01) improvement in their nutritional status.

## Data Availability

Data sharing is not applicable.
